# Signal‐quality‐aware multisensor fusion for atrial fibrillation detection

**DOI:** 10.1049/htl2.12121

**Published:** 2025-02-25

**Authors:** Shane Malone, Barry Cardiff, Deepu John, Arlene John

**Affiliations:** ^1^ School of Electrical and Electronic Engineering University College Dublin Dublin Ireland; ^2^ Biomedical Signals and Systems Group, Faculty of Electrical Engineering, Mathematics and Computer Science University of Twente Enschede Netherlands

**Keywords:** learning (artificial intelligence), medical signal processing

## Abstract

This letter introduces a novel method to enhance atrial fibrillation detection accuracy in healthcare monitoring. Wearable devices often face inconsistent signal quality due to noise. To address this, a multimodal data fusion technique that improves signal reliability during continuous monitoring is proposed. The method improves the precision of detecting R–R intervals by integrating wavelet coefficients from electrocardiogram, photoplethysmogram, and arterial blood pressure signals, weighted according to the quality of each signal. Furthermore, a bi‐directional long short‐term memory network is developed to accurately detect AF based on the derived heartrate or R–R intervals. Unlike prior studies, this work uniquely evaluates the system’s performance under noisy conditions, demonstrating significant accuracy improvements over single‐channel methods. The system's generalizability is confirmed by evaluating the classifier's performance as the number of sensor inputs increases. At a signal‐to‐noise ratio of −10 dB, the accuracy improves by 4.51% with two sensor inputs and by 10.92% with three inputs, compared to using a single input.

## Graphical abstract

This manuscript presents a multisensor fusion algorithm for detecting atrial fibrillation in noisy, ambulatory environments by combining fusing data from electrocardiogram, photoplethysmogram, and arterial blood pressure signals, based on their signal quality. Using wavelet‐based fusion and a bidirectional long‐short‐term memory model, the approach outperforms single‐sensor methods, with better detection performance as the number of sensors increases.

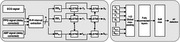

## 1 INTRODUCTION

Continuous patient monitoring is being increasingly utilized in both clinical and ambulatory settings for the early detection of various medical conditions [1]. However, one of the primary challenges in employing these sensors for continuous monitoring is the degradation of signal quality caused by factors such as patient movement, electrode displacement, and baseline drift [2, 3]. To ensure reliable detection of medical conditions, it is essential to develop systems that are robust against these sources of noise. Traditional signal de‐noising methods tend to fail when the signal‐to‐noise ratio (SNR) is low, particularly in the presence of significant motion artefacts that affect wearable devices [4]. In such cases, clinical‐grade devices often return null values, as the estimated signals exceed acceptable thresholds or rely on prior data estimates. To overcome these limitations, incorporating additional signals with higher SNRs is beneficial, as monitoring devices now include a broader range of sensors. A promising technique for such scenarios is multimodal signal fusion–a data processing approach that combines inputs from multiple sources to produce more accurate outcomes than each source individually [5, 6]. This approach enhances overall performance, as the system becomes more reliable with the addition of multiple sensor inputs, each contributing independently [7].

Cardiac disease has a death rate of 32% more than cancer and other diseases, and therefore, early detection is important to reduce mortality [8]. Atrial fibrillation (AF) is the most common arrhythmia, marked by irregular R–R intervals and the absence of a P‐wave, which is replaced by low‐amplitude fibrillatory waves. It was shown that active screening detects more cases than conventional methods in [9]. A 20‐s Holter electrocardiogram (ECG) used in routine primary check‐ups is inadequate for identifying AF, highlighting the need for continuous monitoring. Wearable sensors can provide ongoing active screening, detecting AF without requiring clinic visits [10, 11]. Given the challenges of ensuring noise‐free signals during extended monitoring periods, enhancing the accuracy of early AF detection is crucial for improving patient outcomes. AF detection techniques can be broadly categorized into statistical methods, classical machine learning approaches, and deep learning‐based techniques. The most commonly utilized features for AF classification are inter‐beat intervals (R–R intervals) and the presence of the P‐wave. Many of these approaches rely on features extracted from ECG, photoplethysmogram (PPG), and arterial blood pressure (ABP) signals [12]. In [13], a linear classifier was employed to detect AF events using features derived from R–R intervals. Bi‐directional long short‐term memory (Bi‐LSTM) networks have been shown to achieve high accuracy for AF classification based on R–R intervals in [14]. [15] quantified AF detection was carried out by comparing R–R‐interval time series to normal sinus rhythm (NSR) using an ensemble of templates in [15], and entropy measures were applied to detect AF based on R–R interval irregularity in [16].

In this paper, we aim to extract features relevant to AF detection through the fusion of ECG, PPG, and ABP signals, and demonstrate that classification based on these fused features yields greater accuracy compared to features extracted from individual signals in noisy environments. The novel contributions of this work include: 1. The development of a data fusion technique to enhance AF detection in wearable healthcare systems by integrating wavelet coefficients from ECG, PPG, and ABP signals, weighted based on signal quality. 2. Utilization of a Bi‐LSTM network for fusion and evaluating fusion performance under noisy conditions while demonstrating scalability with multiple sensor inputs. Unlike prior AF classification studies, this work proposes a generalizable algorithm that enables the flexible incorporation of additional sensors tailored to specific use cases.

## 2 METHODOLOGY

### 2.1 Dataset

This work utilises the MIMIC III waveform database matched subset [17], a subset of the MIMIC III database [18]. Since this dataset lacks AF annotations, we utilize publicly available annotations from [19], which were annotated by a board‐certified physician specializing in AF management. The MIT‐BIH atrial fibrillation database [20], commonly used for evaluating AF classifiers, is unsuitable here as it contains only ECG data, whereas the proposed multisensor fusion algorithm uses PPG and ABP signals. Although ABP signals require a bedside monitor with arterial cannulation for acquisition, the ABP signal is included in this study to demonstrate that, under noisy conditions, incorporating additional sensors can enhance detection performance. Therefore, We evaluate our multimodal classifier with the AF‐annotated MIMIC III database (D1) [19]. Additionally, we utilize the MIT‐BIH database (D2) to benchmark our classifier's performance–using ECG data as the sole input–against state‐of‐the‐art classifiers. Both datasets are available through the PhysioNet Physiobank database [21].

### 2.2 Signal quality estimation

Multimodal data fusion can be implemented through various techniques. A simple unweighted fusion of multiple signals results in a static reliance on all signals, even when one or more signals are compromised by noise. As observed in [10], the presence of noise can significantly increase the rate of false positives. To enhance robustness against noise, a weighting scheme based on the relative quality of each signal can be employed, allowing signals of lower quality to have a reduced influence on the fusion process.

**FIGURE 1:**
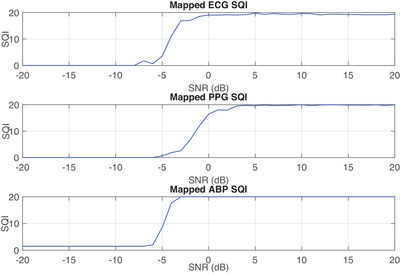
Mapped SQIs for each input signal versus SNR.

In this article, we employ the signal quality index (SQI) technique introduced in [22]. This method utilizes template matching, where the correlation between a pre‐calculated clean signal template and the input signal is used to assess signal quality. The SQI value computed for each input signal sample is then mapped to its corresponding signal‐to‐noise ratio (SNR) using the mapping function described in [22]. This ensures that the SQIs derived from multiple multimodal signals are comparable for similar input SNRs. The resulting set of SQIs, one for each input, varies according to the signal quality and serves as a framework for estimating the input SNR. The mapped SQIs for each signal at different SNR levels are illustrated in Figure 1.

### 2.3 R–R interval extraction

To extract R–R intervals, we use an automated method for heartbeat detection from ECG, PPG, and ABP signals via discrete stationary wavelet transform to capture the relevant time‐frequency components. Although heartbeat events in these three signals are correlated, each signal is analyzed independently to account for the differing effects of noise across signals. This approach aligns with the primary objective of the fusion algorithm, which is to improve atrial fibrillation detection in ambulatory settings. The input signals, sampled at 125 Hz, allow for the use of level 2 and level 3 detail coefficients of the wavelet transform, corresponding to 7.8125–15.625 Hz and 15.625–31.25 Hz, respectively. The stationary wavelet transform is employed to avoid down‐sampling the coefficients at each level, thus preserving time‐domain resolution. The Biorthogonal 6.8 wavelet is selected as the mother wavelet for the ECG signal, and the Symlet 8 wavelet is chosen for PPG signals based on prior studies [5]. The mother wavelet for the ABP signal was determined to be the Biorthogonal 6.8 wavelet through trial and error, as testing with other wavelets showed no significant difference in R‐R interval estimation performance.

**FIGURE 2:**
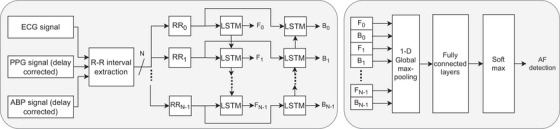
Block diagram for multimodal fusion based system. $F_{n}$ and $B_{n}$ represent outputs from the forward and backward LSTM networks respectively.

We first calculated pulse propagation delays for each patient record by averaging offsets between beats detected in PPG and ABP signals versus ECG. These delays were then applied to align the signals, ensuring that wavelet coefficient peaks were synchronized during fusion. We then fused the level 2 and 3 detail coefficients using a weighted sum based on normalized signal quality indices (SQIs). Beat detection involved identifying local peaks in the fused signal and applying heuristic thresholds to exclude misidentified peaks. The R–R interval sequence was then computed as the time difference between successive peaks [23]. Figure 2 illustrates the block diagram of the proposed system.

### 2.4 Classifier

To generate the feature set from our input signals, we utilize R–R intervals discussed in the previous subsection, which are a standard feature in AF classification [12]. Time‐series transformers and Recurrent Neural Networks (RNNs) are both well‐suited for processing time‐series data. However, while transformers are highly powerful, they demand larger datasets and significant computational resources, making them less practical for our context. As a result, an RNN‐based classifier was opted to handle the R‐R interval sequences efficiently. Long Short‐Term Memory (LSTM) networks, which is a type of RNN, addresses the issue of vanishing gradients. Compared to other RNNs like gated recurrent unit (GRU) and bi‐drectional gated recurrent unit (BGRU), the memory cell mechanism of LSTMs ensures better handling of long‐term dependencies, which is important for the application considered in this work. LSTMs operate by concatenating the input with a hidden state and processing the sequence through three types of gates: forget, input, and output. Each LSTM cell outputs both the cell state and the hidden state, which are then passed to the next cell in the sequence. Our experiments show that 40 hidden units are optimal for classifying R–R intervals in AF detection.

Bi‐directional long short‐term memory (BiLSTM) networks have demonstrated high accuracy in AF classification based on R–R intervals [14]. A BiLSTM network can be conceptualized as consisting of two LSTM networks: one trained on the input sequence in its original order and the other trained on the reversed input sequence. Introduced in [24], BiLSTMs have been shown to enhance classification performance compared to traditional LSTMs. This improvement is attributed to their ability to leverage information from both preceding and subsequent samples relative to each point in the sequence, thereby providing a more comprehensive context for each sample.

To assess various model configurations, we employed the MIT‐BIH database (D2) to enable a direct comparison of our classifier with state‐of‐the‐art models. Optimal classification performance was achieved using sequences of 20 R–R intervals, which provided a balance between effective classification and sufficient time resolution. Given that the average resting heart rate ranges from 60 to 100 beats per minute, a monitoring period of approximately 20 s is sufficient for detecting AF during rest. It is noteworthy that the training dataset contains a substantially higher number of examples of NSR compared to AF instances. To address this imbalance, we use a weighted cross entropy loss function during training.

### 2.5 Evaluation

The extracted feature sets were divided using a standard train‐test split methodology. Due to class imbalance, accuracy alone is not a sufficient metric for evaluating performance. Consequently, we report accuracy, sensitivity, and specificity, specifically for the AF class.

## 3 RESULTS

### 3.1 Testing the classifier with MIT‐BIH AF database

We start by extracting R–R intervals from the MIT‐BIH database (D2), which exclusively contains ECG signals using the wavelet‐based method proposed here. The detection results are presented in Table 1. We compare our model with other AF classifiers that utilize R–R interval features, as outlined in [12]. Our analysis reveals that the accuracy of our model is comparable to that of other AF classifiers when only a single input is used (the ECG signal). However, our model exhibits high sensitivity but comparatively lower specificity.

**TABLE 1:** Comparison with state‐of‐the‐art AF classifiers Based on R–R Intervals on the MIT‐BIH arrhythmia database.

Author	Accuracy	Sensitivity	Specificity
This work	96.55%	97.12%	96.07%
Faust et al. [14]	98.51%	98.32%	98.67%
Henzel et al. [13]	93%	90%	95%
Cui et al. [15]	97.78%	97.04%	97.96%
Islam et al. [16]	96.38%	96.39%	96.38%

### 3.2 Testing on MIMIC III database

**FIGURE 3:**
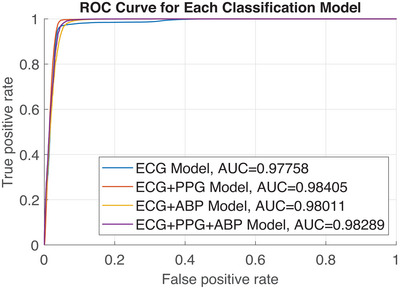
Receiver operator characteristic for each classification model.

We evaluate the performance of multimodal fusion models, which were trained using features extracted from clean signals, on data from the MIMIC III database (D1). For ease of reference, the models are denoted as follows: ECG model ($M_{E}$), PPG model ($M_{P}$), ABP model ($M_{A}$), ECG+PPG model ($M_{E+P}$), ECG+ABP model ($M_{E+A}$), and ECG+PPG+ABP model ($M_{E+P+A}$). The results of these evaluations are presented in Table 2. The data indicate that the fusion‐based models demonstrate superior accuracy compared to the single‐input models. Notably, all models exhibit high sensitivity, as anticipated, due to the weighted loss function prioritizing the detection of AF. The variation in accuracy across the three single signal models arises from differences in signal quality and predictive power for atrial fibrillation detection. This study assumes high‐quality data from the dataset, though this is not always the case. Literature indicates that ECG is the most reliable signal for AF detection, as commonly used by clinicians, surpassing PPG and ABP. However, since PPG and ABP independently provide predictive value, their inclusion in the fusion process can enhance overall reliability, particularly when ECG signal quality is compromised. The reduced accuracy of the single‐input models can be attributed to their lower specificity. Additionally, fusion‐based models, particularly those incorporating the ABP input, show higher sensitivity but lower specificity. Figure 3 displays the receiver operating characteristic (ROC) curves for the models, along with the area under the curve (AUC) for each. A higher AUC indicates superior overall classification performance. Our analysis confirms that the $M_{E+P}$ model demonstrates the highest overall performance. Additionally, the $M_{E+P+A}$ model also surpasses the $M_{E}$ model. These findings suggest that multimodal fusion enhances the accuracy of AF classification in the absence of noise.

**TABLE 2:** Results of testing clean signals from the MIMIC III database.

Model	Accuracy	Sensitivity	Specificity
ECG	94.83%	93.47%	96.49%
PPG	69.50%	83.71%	67.91%
ABP	76.11%	80.75%	68.33%
ECG+PPG	96.20%	98.16%	96.00%
ECG+ABP	95.04%	98.56%	92.77%
ECG+PPG+ABP	95.84%	98.27%	94.43%

**FIGURE 4:**
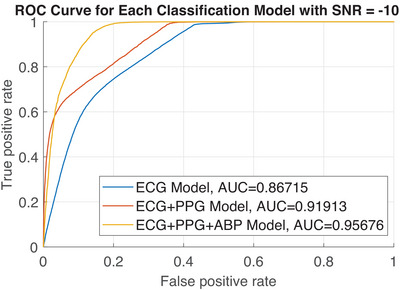
Receiver operator characteristic for each classification model with noise added.

To thoroughly assess the performance of our system under noisy conditions, we selected additive white Gaussian noise for evaluation. This choice is appropriate due to its characteristic of encompassing components across all frequencies, effectively representing a wide range of potential noise scenarios. Furthermore, Gaussian noise facilitates uniform comparison across input signals, which are typically affected by diverse noise types, as the aggregation of multiple independent noise sources tends to approximate a Gaussian distribution. During testing, noise was introduced to 20% of all signals simultaneously at varying signal‐to‐noise ratios. Table 3 presents the results of testing the models with the addition of white Gaussian noise at varying signal‐to‐noise ratios (SNRs) across different input combinations. The results indicate that models exhibit the poorest performance when noise is introduced to the ECG input. Notably, the $M_{E+P+A}$ model maintains significantly higher accuracy under noisy conditions across all inputs compared to the $M_{E+P}$ model. The $M_{E+P+A}$ model is the top‐performing model in most noisy environments; however, it exhibits reduced performance relative to $M_{E+P}$ at signal‐to‐noise ratios (SNRs) below −15 dB. At a signal‐to‐noise ratio (SNR) of −10 dB, the $M_{E+P}$ model exhibits a reduction in sensitivity of 4.54% compared to $M_{E}$, while showing a substantial increase in specificity of 13.75%. Conversely, the $M_{E+P+A}$ model demonstrates an improvement in sensitivity of 9.60% and an increase in specificity of 16.08% relative to $M_{E}$. Figure 4 illustrates the ROC curves for these models when the signals have an input SNR of −10 dB. It is evident that $M_{E}$ has the lowest AUC, thereby confirming that the fusion‐based models outperform it. These results suggest that fusion‐based models experience a lower decrease in accuracy compared to single‐input models in the presence of noise and that the inclusion of a third input further enhances robustness to noise. As discussed previosuly, Table 1 compares the proposed fusion architecture with ECG input to single‐channel algorithms. Due to limited studies, comparisons with other fusion‐based AF detection algorithms are challenging. This work is the first to propose the fusion of ECG, PPG, and ABP signals for AF detection.

**TABLE 3:** Results of testing on noisy signals from the MIMIC III database.

Model	SNR	Accuracy	Sensitivity	Specificity
ECG	0 dB	78.07%	90.95%	63.99%
ECG+PPG		87.53%	74.62%	88.88%
ECG+PPG+ABP		90.97%	97.04%	87.44%
ECG	−5 dB	77.48%	71.24%	77.95%
ECG+PPG		85.15%	78.64%	85.82%
ECG+PPG+ABP		90.89%	97.17%	87.28%
ECG	−10 dB	78.06%	86.41%	68.90%
ECG+PPG		82.57%	81.87%	82.65%
ECG+PPG+ABP		88.98%	96.01%	84.98%
ECG	−15 dB	76.82%	72.17%	77.18%
ECG+PPG		84.23%	81.28%	84.59%
ECG+PPG+ABP		83.61%	89.98%	80.10%
ECG	−20 dB	73.66%	83.08%	69.81%
ECG+PPG		83.12%	87.47%	82.58%
ECG+PPG+ABP		80.12%	80.97%	78.36%

## 4 CONCLUSION

We have developed an atrial fibrillation detection system utilizing signal‐quality‐aware multimodal fusion of ECG, PPG, and ABP signals. Our findings indicate that the fusion‐based systems outperform single‐input systems in terms of detection performance, showcasing the advantages of the proposed method. Furthermore, in the presence of additive white Gaussian noise, the fusion‐based models demonstrate significantly superior performance compared to their single‐input counterparts. The incorporation of additional relevant inputs further enhances performance under noisy conditions. While the single‐channel ECG‐based AF detection algorithm was found to surpass existing state‐of‐the‐art AF detection algorithms, there is evidence suggesting that multimodal fusion could enhance the reliability of continuous health monitoring systems in both hospital and personal wearable settings by improving robustness to noise. Additional research is needed to explore the benefits of fusion, particularly in the presence of motion artefacts A limitation of this work is the exclusive reliance on additive Gaussian noise to evaluate the system’s performance under noisy conditions, as well as not evaluating the performance of the AF detection algorithm on data obtained from wearable devices. Future work should focus on evaluating the fusion algorithm under specific combinations of simulated noise tailored to uniquely impact each individual signal, particularly in the presence of motion artifacts, which are a prevalent source of noise in wearable devices. For practical implementation in wearable devices, future work should address the reduction of computational complexity associated with fusion‐based AF detection, as well as evaluate the performance of the AF detection algorithm on data obtained from wearable sensors. Future investigations should also focus on multi‐class arrhythmia classification and the evaluation of various fusion architectures.

## AUTHOR CONTRIBUTIONS

**Shane Malone**: Formal analysis; investigation; methodology; software; validation; visualization; writing—original draft. **Barry Cardiff**: Funding acquisition; resources; validation; writing—review and editing. **Deepu John**: Funding acquisition; project administration; resources; supervision; writing—review and editing. **Arlene John**: Conceptualization; data curation; formal analysis; methodology; validation; visualization; writing—review and editing.

## CONFLICT OF INTEREST STATEMENT

The authors declare no conflicts of interest.

## Data Availability

The data that support the findings of this study are openly available in Physionet at https://physionet.org/content/mimic3wdb/1.0/.

## References

[1] Saheb, T. , Izadi, L. : The paradigm of IoT big data analytics in the healthcare industry: a review of scientific literature and mapping of research trends. Telematics Inf. 41, 70–85 (2019)

[2] Mohamed Adel, S. , et al.: ECG monitoring systems: review, architecture, processes, and key challenges. Sensors 20(6), 1796 (2020) 32213969 10.3390/s20061796 PMC7147367

[3] Kim, B. , Yoo, S. : Motion artifact reduction in photoplethysmography using independent component analysis. IEEE Trans. Biomed. Eng. 53(3), 566–568 (2006) 16532785 10.1109/TBME.2005.869784

[4] Prabhakararao, E. , Manikandan, M. S. : Efficient and robust ventricular tachycardia and fibrillation detection method for wearable cardiac health monitoring devices. Healthc. Technol. Lett. 3(3), 239–246 (2016) 27733933 10.1049/htl.2016.0010 PMC5047284

[5] John, A. , et al.: A multimodal data fusion technique for heartbeat detection in wearable IoT sensors. IEEE Internet Things J. 9(3), 2071–2082 (2021)

[6] Hall, D.L. , Llinas, J. : An introduction to multisensor data fusion. Proc. IEEE 85(1), 6–23 (1997)

[7] John, A. , et al.: Multimodal multiresolution data fusion using convolutional neural networks for wearable sensing. IEEE Trans. Biomed. Circuits Syst. 15(6), 1161–1173 (2021) 34882563 10.1109/TBCAS.2021.3134043

[8] Sun, J. : Automatic cardiac arrhythmias classification using CNN and attention‐based RNN network. Healthc. Technol. Lett.. 10(3), 53–61 (2023) 37265837 10.1049/htl2.12045 PMC10230559

[9] Fitzmaurice, D.A. , et al.: Screening versus routine practice in detection of atrial fibrillation in patients aged 65 or over: cluster randomised controlled trial. BMJ 335(7616), 383–383 (2007) 17673732 10.1136/bmj.39280.660567.55 PMC1952508

[10] Giebel, G.D. , Gissel, C. : Accuracy of mHealth devices for atrial fibrillation screening: systematic review. JMIR mHealth uHealth 7(6), e13 641–e13 641 (2019) 10.2196/13641 PMC6598422 31199337

[11] Turakhia, M.P. , et al.: Rationale and design of a large‐scale, app‐based study to identify cardiac arrhythmias using a smartwatch: the apple heart study. Am Heart J. 207, 66–75 (2019) 30392584 10.1016/j.ahj.2018.09.002 PMC8099048

[12] Faust, O. , Ciaccio, E.J. , Acharya, U.R. : A review of atrial fibrillation detection methods as a service. Int. J. Environ. Res. Public Health 17(9), 3093 (2020) 32365521 10.3390/ijerph17093093 PMC7246533

[13] Henzel, N. , Wróbel, J. , Horoba, K. : Atrial fibrillation episodes detection based on classification of heart rate derived features. In: 2017 MIXDES ‐ 24th International Conference “Mixed Design of Integrated Circuits and Systems”, Conference Proceedings, pp. 571–576. IEEE, Piscataway, NJ (2017)

[14] Faust, O. , et al.: Automated detection of atrial fibrillation using long short‐term memory network with RR interval signals. Comput. Biol. Med. 102, 327–335 (2018) 30031535 10.1016/j.compbiomed.2018.07.001

[15] Cui, X. , et al.: Automated detection of paroxysmal atrial fibrillation using an information‐based similarity approach. Entropy 19(12), (2017)

[16] Islam, M.S. , et al.: Rhythm‐based heartbeat duration normalization for atrial fibrillation detection. Comput. Biol. Med. 72, 160–169 (2016) 27043858 10.1016/j.compbiomed.2016.03.015

[17] Moody, B. , et al.: MIMIC‐III waveform database matched subset. https://physionet.org/content/mimic3wdb‐matched/1.0/ (2020). Accessed 1 Dec 2024

[18] Johnson, A.E.W. , et al.: MIMIC‐III, a freely accessible critical care database. Sci. Data 3(1), 160035 (2016) 27219127 10.1038/sdata.2016.35 PMC4878278

[19] Bashar, S.K. , et al.: Atrial fibrillation detection during sepsis: study on MIMIC III ICU data. IEEE J. Biomed. Health. Inf. 24(11), 3124–3135 (2020) 10.1109/JBHI.2020.2995139 PMC7670858 32750900

[20] Moody, G. , Mark, R. : A new method for detecting atrial fibrillation using R‐R intervals. Comput. Cardiol. 10, 4 (1983)

[21] Goldberger, A. , et al.: Components of a new research resource for complex physiologic signals. Circulation 101(23), 215–220 (2000) 10.1161/01.cir.101.23.e215 10851218

[22] John, A. , Cardiff, B. , John, D. : A generalized signal quality estimation method for IoT sensors. In: 2020 IEEE International Symposium on Circuits and Systems (ISCAS), Conference Proceedings, pp. 1–5. IEEE, Piscataway, NJ (2020)

[23] John, A. , et al.: An evaluation of ECG data fusion algorithms for wearable IoT sensors. Inf. Fusion 96, 237–251 (2023)

[24] Schuster, M. , Paliwal, K.K. : Bidirectional recurrent neural networks. IEEE Trans. Signal Process. 45(11), 2673–2681 (1997)

